# Chemotherapy-Induced Peripheral Neuropathy in Cancer Patients: A Four-Arm Randomized Trial on the Effectiveness of Electroacupuncture

**DOI:** 10.1155/2013/349653

**Published:** 2013-08-28

**Authors:** M. Rostock, K. Jaroslawski, C. Guethlin, R. Ludtke, S. Schröder, H. H. Bartsch

**Affiliations:** ^1^Tumor Biology Center at the Albert Ludwigs University Freiburg, 79106 Freiburg, Germany; ^2^Institute of Complementary Medicine, University Hospital Zurich, 8006 Zurich, Switzerland; ^3^Hubertus Wald Tumor Center, University Cancer Center Hamburg, 20246 Hamburg, Germany; ^4^University Medical Center Freiburg, 79106 Freiburg, Germany; ^5^Institute of General Practice, Johann Wolfgang Goethe University, 60590 Frankfurt/M, Germany; ^6^Carstens Foundation Essen, 45276 Essen, Germany; ^7^Hanse Merkur Center for Traditional Chinese Medicine at the University Medical Center Hamburg-Eppendorf, 20246 Hamburg, Germany

## Abstract

*Purpose*. Chemotherapy-induced peripheral neuropathy (CIPN) is a common and dose-limiting side effect of cytostatic drugs. Since there are no proven therapeutic procedures against CIPN, we were interested to define the role of electroacupuncture (EA) from which preliminary data showed promising results. *Methods*. In a randomized trial with a group sequential adaptive design in patients with CIPN, we compared EA (LV3, SP9, GB41, GB34, LI4, LI11, SI3, and HT3; *n* = 14) with hydroelectric baths (HB, *n* = 14), vitamin B1/B6 capsules (300/300 mg daily; VitB, *n* = 15), and placebo capsules (*n* = 17). The statistical power in this trial was primarily calculated for proving EA only, so results of HB and VitB are pilot data. *Results*. CIPN complaints improved by 0.8 ± 1.2 (EA), 1.7 ± 1.7 (HB), 1.6 ± 2.0 (VitB), and 1.3 ± 1.3 points (placebo) on a 10-point numeric rating scale without significant difference between treatment groups or placebo. In addition no significant differences in sensory nerve conduction studies or quality of life (EORTC QLQ-C30) were found. *Conclusions*. The used EA concept, HB, and VitB were not superior to placebo. Since, contrary to our results, studies with different acupuncture concepts showed a positive effect on CIPN, the effect of acupuncture on CIPN remains unclear. Further randomized, placebo controlled studies seem necessary. This trial is registered with DRKS00004448.

## 1. Background

Chemotherapy-induced peripheral neuropathy (CIPN) is common and can be dose limiting for several cytotoxic drugs, for example, the antitubulins (paclitaxel, docetaxel, ixabepilone, and vincristine), platinum analogs (cisplatin, carboplatin, and oxaliplatin), and the proteasome inhibitors bortezomib and thalidomide. CIPN symptoms usually appear symmetrically in a stocking-glove shaped distribution pattern. Typical symptoms include numbness and tingling, whereas neuropathic pain appears less frequently. Affected patients experience considerable impairments including difficulty in walking, increased risk of falls, and weakness and restrictions in fine motor skills such as writing and other differentiated manual tasks. After the completion of chemotherapy, the symptoms frequently determine the patient's quality of life and often considerably hinder social rehabilitation, social reintegration, and return to work [1, 2].

Various substances including amifostine, glutathione, vitamin E, glutamic acids, intravenous calcium and magnesium infusions, and neurotrophic growth factors have been examined in clinical studies as a prophylaxis against chemotherapy-induced neurotoxic effects. No studies have shown convincing evidence of substantial clinical benefit [3, 4]. Anticonvulsants (e.g., carbamazepine and in particular gabapentin), tricyclic antidepressants (e.g., amitriptyline), and selective serotonin reuptake inhibitors (e.g., venlafaxine) play a clinical role in the prevention and treatment of neuropathic pain. However, these drugs are ineffective for the treatment of the typical sensory CIPN symptoms and cannot induce neuroprotection or neuroregeneration [5–8].

A recently published survey revealed that approximately 43% of patients with chronic peripheral neuropathy use or have used complementary or alternative medicine (CAM), including high doses of vitamins, magnet therapy, herbal remedies, and chiropractic treatment. In addition, 30% of patients used acupuncture. Approximately a quarter of the patients stated that their symptoms improved after using CAM therapies [9].

The WHO and leading German acupuncture societies have long-listed neuropathy as an indication for acupuncture [10]. Few reports have published on its effect. Several studies demonstrated beneficial effects of acupuncture in diabetic neuropathy [11–14], HIV-associated neuropathy [15, 16], and in peripheral neuropathy with an unclear etiology [17]. The very limited conclusions from these studies resulted either from the small number of treated patients, the uncontrolled study design, or the publications in Chinese journals with merely an abstract in English and therefore not easy to evaluate. Acupuncture in combination with electrostimulation for treating CIPN has rarely been evaluated [18, 19]; however it has been demonstrated to improve neuropathic pain in paclitaxel-treated rats at both low (10 Hz) and high (100 Hz) frequencies [20].

In German-speaking countries, hydroelectric baths are traditionally used in many rehabilitation centers for the treatment of peripheral neuropathy [21]. However, their effectiveness has not been proven via controlled studies. The use of vitamin B supplements to treat neuropathy is also very common but also without rigorous clinical evaluation [22].

In this randomized controlled trial we compared the effects of electroacupuncture with placebo, hydroelectric baths, and vitamin B complex.

## 2. Methods

### 2.1. Study Design

The study was conducted as a randomized, placebo-controlled trial comparing electroacupuncture (EA) with hydroelectric baths (HB), high doses of vitamin B1 and B6 (VitB), and a placebo for the treatment of CIPN. Patients and physicians were blind to the VitB and placebo treatment but not to EA or HB.

The trial was planned according to a group sequential adaptive design with one interim analysis [23]. The experimental type I error rate was set at a one-sided *α* = 0.025. Early cessation was planned if either EA proved to be significantly better than placebo (i.e., *P* < *α*_1_ = 0.0207) or showed no relevant superiority (*P* > *α*_0_ = 0.6). 

Patients were allocated to one of the four treatment groups by a nonstratified block randomization with randomly varying block lengths. The biometrician drew numbers from the “ranuni” random number generator of the SAS/STAT software and prepared sealed, opaque, and sequentially numbered envelopes containing the treatment assignments. If a patient fulfilled all inclusion criteria the study physician opened the lowest numbered envelope to reveal the patient's assignment, that is, “EA,” “HB,” or “medical intervention.” Patients from the latter group were given coded bottles of study capsules that were prepared prior to the beginning of the study by an impartial pharmacist. The bottles contained 63 capsules of VitB or placebo. Placebo and VitB capsules were identical in form, taste, and odour. The randomization list was kept closed by the biometrician, the pharmacist, and the principle investigator and was not accessible to the study physician.

The study was conducted according to the Declaration of Helsinki and its amendments. The study protocol was approved and accepted by the Ethics Committee of the Albert Ludwigs-University of Freiburg. 

### 2.2. Patients

The study enrolled male and female adult cancer patients, who were in remission after chemotherapy with taxanes, platinum derivatives, or vinca alkaloids and who presented with symptoms of CIPN. Patients were recruited from a 3-4-week inpatient rehabilitation program in the Tumor Biology Center at the Albert Ludwigs University Freiburg, Germany. All patients received detailed information about the study and provided written informed consent before participation.

### 2.3. Treatments

Treatment protocols spanned 3 weeks and were as follows.

#### 2.3.1. Electroacupuncture (EA)

8 ± 1 sessions of EA were scheduled to treat the affected extremities with the following point combination: LV3 (Taichong), SP9 (Xiongxiang), GB41 (Zulingqi), GB34 (Yanglingquan) (legs; in patients with CIPN symptoms in the lower extremities) and LI4 (Hegu), LI11 (Quchi), SI3 (Houxi), and HT3 (Shaohai) (arms; in patients with CIPN symptoms in the upper extremities). Patients with CIPN symptoms in the upper and lower extremities were treated with the complete point combination. According to the practices of traditional Chinese medicine (TCM) the acupuncture needles were deeply inserted bilaterally until the deqi phenomenon (sensation which spreads over the whole body part described as “aching,” “soreness,” “pressure,” or “tingling” [24]) was triggered. Each session included 15 minutes of electrostimulation (50 Hz) consisting of a combination of rectangular currents and high amplitude waves. The stimulus strength was increased until the deqi phenomenon was triggered again. The acupuncture was carried out by two specially trained, highly experienced physicians at the University Medical Center Freiburg who had completed training in acupuncture with the German Physicians Association.

#### 2.3.2. Hydroelectric Baths (HB)

8 ± 1 sessions of HB were scheduled to treat the affected extremities. The patients dipped their arms up to a hand's width above the elbow and their feet up to a hand's width above the ankle into a special water basin with water at a temperature of about 35°C. The water served as an electrode for the skin surface. Each treatment lasted for 15 minutes with cross-galvanisation of each individual extremity by low-frequency (50 Hz) faradic current (direct current impulses) up to the individual's sensitive threshold (i.e., the point where the tingling feeling is considered to be just acceptable).

#### 2.3.3. Vitamin B Complex (VitB)

 The treatment consisted of 3 capsules of high-dosage vitamin B1/B6 (100 mg thiamine nitrate, 100 mg pyridoxine hydrochloride) per day for three weeks. 

#### 2.3.4. Placebo

The placebo treatment consisted of 3 lactose capsules per day identical in form, taste, and odour to the VitB capsules. 

## 3. Outcomes

### 3.1. Primary Outcome

By means of detailed questionnaires, patients were interviewed before the start of the therapy (day 0), after treatment on day 21, and during follow-up on day 84, about extension and intensity (non, mild, moderate, or severe) of their CIPN complaints (numbness, swelling, tingling, pain, and subjective impairment in everyday life and at work).

Finally, patients were asked to describe how heavily they suffered at the respective point in time from CIPN complaints altogether and to rate the severity of neuropathic symptoms on a numerical rating scale (NRS)—ranging from 0 (no complaints) to 10 (highest imaginable complaints). The change from day 0 to day 21 on this patient-reported numerical rating scale was the primary outcome of the study.

### 3.2. Secondary Outcomes

Before the start of the study (day 0) and after completion of the treatment (day 21), the patients were examined by an independent neurologist. The neurologist ascertained a neuropathy score and performed electroneurographical tests.

The neuropathy score (0–15 points) was based on sensory symptoms (0–3 points), pin sensibility (0–3 points), vibratory threshold (0–3 points), strength (0–3 points), and deep tendon reflexes (0–3 points). The electroneurographical tests included sensible nerve conduction studies of the median (upper extremities) and the sural nerve (lower extremities). 

Finally, the neurologist evaluated the intensity of the CIPN complaints by classification according to the NCI common toxicity criteria (CTC) [25].

Examinations by the neurologist were only performed at day 0 and day 21, while the follow-up interview after 12 weeks, at day 84, was done in writing, via questionnaires sent to the patients' homes all over Germany.

Quality of life: the study participants' quality of life was examined at day 0, day 21, and day 84 by means of EORTC QLQ-C30 [26].

## 4. Statistics

This trial was planned using a group-sequential adaptive design which allowed for an adaptation (i.e., recalculation) of sample sizes after the first interim analysis. A priori power calculation showed that our test procedure had a type II error probability to stop the trial early for nonsuperiority (i.e., *P* > *α*_0_ = 0.6) of 16.7% (type II error) if EA had a moderate effect beyond the placebo of 0.5 standard deviations.

The data were analyzed using ANCOVA and modeling the treatment group and the baseline value (linear predictor) as covariates. Within this model, treatment groups were compared in pairs by one-sided *t*-tests following the principle of a priori ordered hypothesis [27]. All six comparisons were ordered according to a previously defined list which started with the comparison of EA and placebo. This list was processed successively and a subsequent comparison was performed if and only if the actual comparison could be rejected at the nominal level (i.e., 0.0207 at the interim analysis). This procedure ensured that the overall experimental type I error rate was maintained.

All analyses were performed based on intention to treat; that is, all randomized patients who received at least one study treatment were included in all (effectiveness or safety) analyses. Missing values were imputed using last observation carried forward [28].

## 5. Results

**Figure 1:**
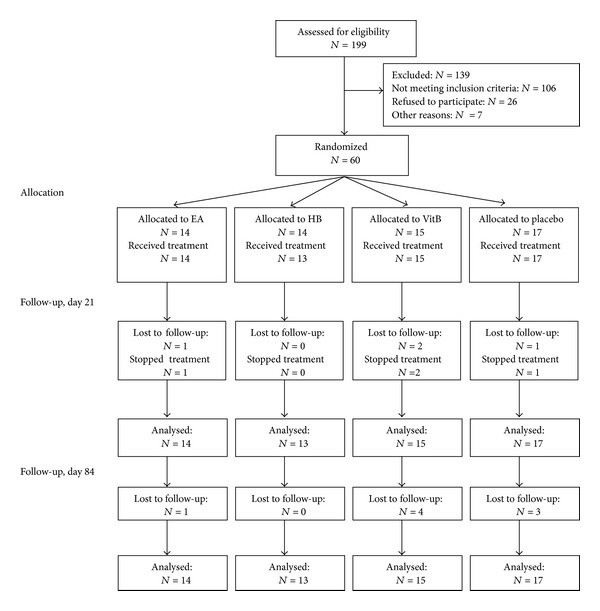
Flow chart.

From September 2000 to February 2003 a total of 199 cancer patients with CIPN were assessed for eligibility and 60 were randomized into one of the four treatment groups (Figure 1). The main reasons for exclusion were pretreatment with vitamin B (*N* = 40), progressive cancer (*N* = 22), ongoing chemotherapy (*N* = 16), treatment with cytostatic drugs not allowed in the protocol (*N* = 17), or patient's unwillingness to take part (*N* = 26). Immediately after randomization one patient in the HB group withdrew his consent (before receiving any study treatment). 4 patients stopped treatment: 1 EA patient stated he was anxious of being needled (day 1), 1 VitB patient's tumor progressed (day 13), 1 placebo patient withdrew his consent (day 1), and 1 VitB patient found the study too much strain (day 1). Another 4 patients were lost to follow-up after day 21 without providing any reason. 

*(a) Baseline Data. *The majority of patients were female (78%) and were on average 52.7 ± 10.0 years old, and 92% had ECOG performance status of 0. Nineteen patients were obese (BMI > 30). Four patients (3 HB, 1 placebo) presented with additional neurological problems other than CIPN, 2 patients had facial paresis, one patient had double vision, and one had diminished strength in the right hand. Overall, the groups were balanced with regard to demographic characteristics, health status, and comorbidities (Table 1).

The underlying cancer diagnoses as well as the cancer treatments were very heterogeneous within the study cohort (Table 2). Seventeen patients had a lymphoma (4 Hodgkin and 13 non-Hodgkin), and 42 patients had solid tumors, predominantly breast cancer (21) and ovarian cancer (13). Breast cancer was the most frequent disease in the placebo group and lymphoma in the HB group. Due to these imbalances there were also some differences in the use of chemotherapeutic drugs: vinca alkaloids were most often used in the VitB group and taxanes in the EA and the placebo groups, but these differences were statistically not significant (all *P* values > 0.30, chi-square tests). Moreover, the number of chemotherapy courses in total, or with neurotoxic chemotherapy only, was comparable between the groups (*P* = 0.291 and *P* = 0.667, Kruskal-Wallis tests). 

The mean time since first cancer diagnosis ranged from 12.1 months in the EA to 24.9 months in the HB group. As the latter was dominated by an extreme exception (112 months) these differences were not statistically relevant (*P* = 0.825, Kruskal-Wallis test). Similarly, mean times from the last chemotherapy, last surgery, or last radiotherapy were comparable between groups (all *P* values >0.25). 

Only a few patients (*N* = 7; 11.9%) reported that their first CIPN complaints had occurred after the chemotherapy was finished; mostly, complaints were first noticed during chemotherapy. Symptoms usually started during the first or second chemotherapy cycle. At the start of the study, one-third of the patients had been experiencing CIPN for more than 6 months (*N* = 20; 33.9%) and another two-fifths had it for more than 3 months (*N* = 25; 42.4%). In 31 patients the CIPN had been stable over the last 4 weeks (52.5%), in 23 patients it had improved (38.9%), and in 4 patients it had worsened (6.8%). The treatment groups were balanced with respect to all these parameters (all *P* values >0.15, Kruskal-Wallis tests). 

**Figure 2:**
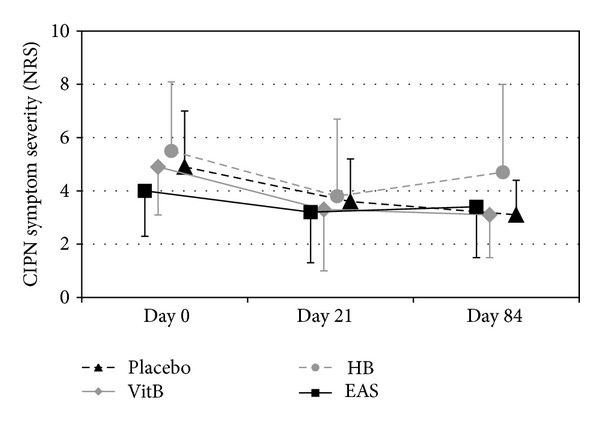
CIPN perceived symptom severity day 0, day 21, and day 84 (severity rated on a 10-point numerical rating scale (NRS); all values are mean ± SD).

Although statistically not significant (*P* = 0.263, Kruskal-Wallis test), mean baseline symptoms varied considerably between groups and ranged from 4.0 ± 1.7 in the EA to 5.5 ± 2.6 in the HB group (Table 4 and Figure 2). At study entry subjective CIPN complaints of the included patients differed as shown in Table 3: while every patient in EA and HB groups suffered from parasthesia, only three-quarters in vitamin B group and 88% in placebo group did so. In the placebo group less patients described painful neuropathy compared to the treatment groups. Subjective impairment in fine motor skills was nearly equal in all four groups. 

*(b) Treatment Results.* Symptoms improved similarly in all groups during the three weeks of treatment and remained at this lower level for another 9 weeks, except in the HB group where some deterioration was observed. At day 21, improvement was best in the HB (1.7 ± 1.7) and the VitB group (1.6 ± 2.0). Compared to placebo (1.3 ± 1.3) EA showed worse effects (0.8 ± 1.2) resulting in a group difference of *d* = −0.3 (CI: −1.4 to 0.8; *P* = 0.705). As the *P* value exceeded the predefined threshold of *α*_0_ = 0.6, the study was stopped early at the first interim analysis. Moreover, improvements in the EA group were smaller than in the VitB group (*d* = −0.5; CI: −1.7 to 0.6) and the HB group (*d* = −0.2; CI: −1.3 to 0.9) (Table 4).

The neuropathy score decreased in all groups during treatment to a similar degree. Improvements were observed most frequently in the EA group and were smallest in the HB and placebo groups (Table 5). Group differences were not significant between any two groups; for example, the difference between EA and placebo was *d* = −0.4 (CI: −1.1 to 0.3; *P* = 0.128). 

There were no statistically significant differences between the treatment groups by electroneurographical test results (Table 6) and NCI common toxicity criteria classification (Table 7). Sensory neuropathy symptoms improved similarly in all four groups: 32.7% with a CTC grade 2 or 3 at day 0 and 17.3% at day 21; 0% with a CTC grade 0 at day 0 and 21.2% at day 21. 

Health related quality of life also moderately improved in all groups, but without any statistical group differences at day 21 (Table 8). 

## 6. Discussion

In this randomized, placebo-controlled study, electroacupuncture, hydroelectric baths, and a high dosage of oral vitamin B1/B6 combination were studied to determine their effectiveness and safety in patients with chemotherapy-induced peripheral neuropathy. Sixty cancer patients were included within the setting of an inpatient oncology rehabilitation program.

In our study we observed no therapeutic advantage of electroacupuncture over an orally administered placebo control. In addition, no effects of hydroelectric baths and vitamin B1/B6 were demonstrated. It should, however, been taken into consideration that the statistical power of the later tests was low because the study was powered to test the effectiveness of electroacupuncture.

When evaluating our results, several limitations must be considered. Unexpectedly, the intensity of CIPN complaints at baseline was relatively low, particularly in the electroacupuncture group. Our study design, including sample size calculations, was based on experiences with patients with more severe CIPN symptoms and higher pain scores showing larger clinical effects of electroacupuncture treatment. Consequently, there were no large margins for many patients to improve considerably, and our study results might have been influenced by a floor effect. 

In spite of the limitation of this study, we are convinced that reporting negative results is of importance. Studies reporting positive results are more likely to be published [29], while negative results more often have to be published in journals with lower impact factors [30]. By publishing our negative results, we hope our data will have an impact on the critical discussion on study designs and acupuncture concepts for CIPN.

In 2006, a small case series (*n* = 5) reported encouragingly positive results on acupuncture without electrical stimulation for the treatment of CIPN [31]. All five patients showed improvements in pain, numbness, and tingling. In contrast to our study, all patients suffered from painful peripheral neuropathy with high symptom scores and were treated over a time period of 12 weeks. The authors pointed out that they are carrying out a larger trial, the results of which are eagerly awaited. A randomized controlled trial on acupuncture for the treatment of CIPN without electrical stimulation was conducted in Beijing, China. The authors describe significant better treatment effects in the acupuncture group in relation to the control group treated with adenosylcobalamin [32]. A recently published small pilot study on acupuncture for the treatment of CIPN (*n* = 11) reports improvements in nerve conduction studies as well as in the subjective rating of the patients [33].

Several different acupuncture concepts have been used in the treatment of CIPN. Different to our protocol, other studies that reported positive effects of acupuncture treatments in CIPN used local points on the extremities like EX-LE 10 (Bafeng), EX-UE 9 (Baxie), and EX-LE 12 (Qiduan) [31, 33–35] or ear acupuncture for neuropathic pain [36]. In particular the local points on the affected limb/region might activate local, segmental, and spinal and central reflexes in accordance with results on animal models [20].

In traditional Chinese medicine an individualized approach with personalized acupuncture treatment is usually expected, while in acupuncture studies there is the necessity of standardization of the procedures, as done in this study. So our results only indicate that our particular standardized acupuncture protocol might not be effective in the treatment of CIPN, but the results cannot be generalized to other acupuncture concepts.

This study was embedded in a routine care rehabilitation program delivered at a specialized clinic in Southwest Germany. Participating patients came from many regions in Germany and thus were only available for a three-week treatment and observation period. Considering the frequent long-term chronic course of the CIPN symptoms it cannot be excluded that a longer treatment period might have yielded different results for electroacupuncture or one of the other therapies under study. Prior studies on acupuncture treatment in peripheral neuropathy have shown that measurable results can only be found after a longer period of treatment up to 10 weeks [17]. While peripheral neuropathy is a disease with structural damage of the nerves, any stable successful treatment has to induce neuroregeneration. The time of functional recovery varies, ranging usually from 3 to 6 months, depending on the level of the lesion and factors in regeneration [37]. So effects of treatment naturally take longer than in other indications of acupuncture treatment with functional states like in pain or vegetative imbalance.

All participating patients were not treated exclusively by the therapies under study but concomitantly received various medical and psychological interventions, depending on the individual need of each patient. This included regular participation in sport therapy sessions, psychoeducative groups, art therapies, ergotherapies, relaxation methods, physiotherapies, massages, and lymph drainages. Effects of these interventions may have contributed to a remarkable effect in the placebo group—as well as to all other groups—thus possibly diminishing the estimated group differences. Positive effects of this program—as delivered in the study center—have already been demonstrated [38]. 

Nevertheless further studies are necessary, to evaluate the role of acupuncture in the treatment of CIPN. The use of nerve conduction studies as an objective parameter for the evaluation of treatment effects [17] as well as recently introduced better outcome measures for the standardization of studies of CIPN will be helpful to improve the quality of future studies [39].

## 7. Conclusion

A specific standardized electroacupuncture concept, as well as vitamin B1/B6 and hydroelectric baths, showed similar treatment effects on CIPN and was not superior to placebo control. While contrary to our results other studies with different acupuncture concepts and longer treatment periods showed a positive effect on CIPN, the effect of acupuncture on CIPN remains unclear. Further randomized, placebo controlled studies seem necessary.

## Figures and Tables

**Table 1:** Basic data (no. of patients or mean ± SD).

	EA	HB	VitB	Placebo
Sex (m : f)	4 : 10	1 : 12	5 : 10	3 : 14
Age (years)	49.9 ± 9.6	52.3 ± 11.3	56.3 ± 11.1	52.0 ± 8.1
Body mass index (kg/m²)	24.1 ± 4.1	25.8 ± 5.5	24.5 ± 3.3	26.1 ± 4.9
General condition (ECOG score 0)	14 (100%)	11 (84.6%)	14 (93.3%)	15 (88.2%)
Neurological problems (other than CIPN)	0 (0.0%)	3 (23.1%)	0 (0.0%)	1 (5.9%)

**Table 2:** History of cancer and CIPN (no. of patients or mean ± SD).

	EA	HB	VitB	Placebo
Breast cancer	6 (42.9%)	3 (23.1%)	4 (26.7%)	8 (47.1%)
Ovarian cancer	3 (21.4)	3 (23.1%)	4 (26.7%)	3 (17.6%)
Other	1 (7.1%)	2 (15.4%)	1 (6.7%)	4 (23.5%)
Lymphoma	4 (28.6%)	5 (38.5%)	6 (40.0%)	2 (11.8%)
Time from first diagnosis (months)	12.1 ± 11.6	24.9 ± 38.6	14.0 ± 13.8	14.5 ± 13.7
Secondary cancer	2 (14.3%)	3 (23.1%)	4 (26.7%)	4 (23.5%)
Chemotherapy				
Vinca alkaloids	4 (28.6%)	5 (28.5%)	6 (40.0%)	3 (17.6%)
Platin derivatives alone	1 (7.1%)	2 (15.4%)	0 (0.0%)	3 (17.6%)
Taxanes alone	6 (42.9%)	3 (23.1%)	4 (26.7%)	8 (47.1%)
Platin derivatives and taxanes combined	3 (21.4%)	3 (23.1%)	5 (33.3%)	5 (29.4%)
Total no. of different cytostatics	2.1 ± 1.4	1.5 ± 0.9	1.7 ± 1.0	2.0 ± 0.8
No. of diff. neurotoxic cytostatics only	1.6 ± 1.2	1.1 ± 0.3	1.2 ± 0.4	1.3 ± 0.6
Time from last chemotherapy (weeks)	20.1 ± 27.4	27.8 ± 49.3	8.7 ± 5.8	14.2 ± 17.0
Cancer surgery	11 (78.6%)	6 (46.2%)	10 (66.7%)	15 (88.2%)
Radiotherapy	9 (64.3%)	8 (61.5%)	7 (46.7%)	13 (76.5%)
Duration of CIPN > 6 months	6 (42.9%)	5 (38.5%)	5 (33.5%)	4 (23.5%)
CIPN stable or worsened	9 (64.3%)	7 (53.8%)	9 (60.0%)	10 (58.8%)

**Table 3:** CIPN: detailed subjective complaints.

Symptoms	EA	HB	VitB	Placebo	Sum
	*N* = 14 (%)	*N* = 13 (%)	*N* = 15 (%)	*N* = 17 (%)	*N* = 59 (%)
Numbness	11 (78.6%)	10 (76.9%)	13 (86.7%)	16 (94.1%)	50 (84.7%)
Sensation of swelling	9 (64.3%)	10 (76.9%)	8 (53.3%)	12 (70.6%)	39 (66.1%)
Parasthesia	14 (100%)	13 (100%)	11 (73.3%)	15 (88.2%)	53 (89.8%)
Pain	7 (50%)	6 (46.2%)	6 (40%)	2 (11.8%)	21 (35.6%)
Subjective impairment in walking	7 (50%)	8 (61.6%)	9 (60.0%)	14 (82.4%)	38 (64.4%)
Subjective impairment in fine motor skills	7 (50%)	8 (61.6%)	10 (66.7%)	10 (58.9%)	35 (59.3%)

**Table:** (a)

Group	Day	Mean ± SD	Diff. mean day 21–day 0 day 84–day 0
EA (*N* = 14)	0	4.0 ± 1.7	
	21	3.2 ± 1.9	**−0.8**
	84	3.4 ± 1.9	−0.6

HB (*N* = 13)	0	5.5 ± 2.6	
	21	3.8 ± 2.9	**−1.7**
	84	4.7 ± 3.3	−0.8

VitB (*N* = 15)	0	4.9 ± 1.8	
	21	3.3 ± 2.3	**−1.6**
	84	3.1 ± 1.6	−1.8

Placebo (*N* = 17)	0	4.9 ± 2.1	
	21	3.6 ± 1.6	**−1.3**
	84	3.1 ± 1.3	−1.8

**Table:** (b)

	Difference	95% CI	One-sided *t*-test	Two-sided *t*-test
EA versus placebo	0.3	−0.8–1.4	*P* = 0.705	*P* = 0.59
HB versus placebo	−0.2	−1.3–0.9	*P* = 0.35	*P* = 0.699
EA versus VitB	0.5	−0.6–1.7	*P* = 0.83	*P* = 0.34
HB versus VitB	0.0	−1.1–1.2	*P* = 0.52	*P* = 0.959
EA versus HB	0.2	−0.9–1.3	*P* = 0.65	*P* = 0.699
VitB versus placebo	−0.2	−1.3–0.8	*P* = 0.323	*P* = 0.646

**Table 5:** Neuropathy score (mean ± SD).

	Group	Day 0	Day 21	Change day 0 to 21
Neuropathy score (0–15)	EA	4.5 ± 1.5	3.5 ± 1.2	−1.0 ± 1.1
	HB	5.0 ± 2.2	4.4 ± 2.1	−0.6 ± 0.9
	VitB	3.9 ± 1.2	3.2 ± 1.3	−0.7 ± 0.8
	Placebo	4.1 ± 1.5	3.5 ± 1.7	−0.6 ± 1.0

**Table 6:** Electroneurographical tests (amplitude and nerve conduction velocity) day 21–day 0 (mean ± SD).

Group	Day	Sural nerve		Median nerve	
		Amplitude (norm > 10 *μ*V)	NCV (norm > 42 m/s)	Amplitude (norm > 7 *μ*V)	NCV (norm > 45 m/s)
EA (*N* = 13)	0	5.4 ± 3.6	46.4 ± 4.7	19.0 ± 10.7	48.2 ± 4.7
	21	7.4 ± 6.0	46.0 ± 3.7	16.8 ± 7.0	48.4 ± 4.4
Diff. mean day 21–day 0		*+2.0 *	*−0.4 *	*−2.2 *	*+0.2 *

HB (*N* = 12)	0	4.3 ± 2.2	46.2 ± 3.4	15.5 ± 7.8	40.7 ± 9.7
	21	6.3 ± 2.6	46.8 ± 7.1	15.9 ± 9.8	46.3 ± 6.3
Diff. mean day 21–day 0		*+2.0 *	*+0.6 *	*+0.4 *	*+5.6 *

VitB (*N* = 13)	0	3.9 ± 2.7	45.0 ± 4.3	15.9 ± 9.8	46.9 ± 4.1
	21	5.1 ± 3.8	45.0 ± 3.8	15.3 ± 6.4	51.2 ± 3.1
Diff. mean day 21–day 0		*+1.2 *	*±0 *	*−0.6 *	*+4.3 *

Placebo (*N* = 14)	0	5.3 ± 3.1	45.3 ± 4.2	17.5 ± 9.5	47.1 ± 7.5
	21	6.3 ± 3.4	45.9 ± 5.8	16.8 ± 8.5	50.6 ± 6.0
Diff. mean day 21–day 0		*+1.0 *	*+0.6 *	*−0.7 *	*+3.5 *

**Table 7:** NCI common toxicity criteria (sensory item) day 21–day 0.

Group	Day	CTC sensory item			
		0	1	2	3
		*N* (%)	*N* (%)	*N* (%)	*N* (%)
EA (*N* = 13)	0	0 (0%)	10 (76.9%)	3 (23.1%)	0 (0%)
	21	2 (15.4%)	9 (69.2%)	2 (15.4%)	0 (0%)
HB (*N* = 12)	0	0 (0%)	4 (33.3%)	6 (50.0%)	2 (16.7%)
	21	2 (16.7%)	7 (58.3%)	2 (16.7%)	1 (8.3%)
VitB (*N* = 13)	0	0 (0%)	11 (84.6%)	2 (15.4%)	0 (0%)
	21	3 (23.1%)	9 (69.2%)	1 (7.7%)	0 (0%)
Placebo (*N* = 14)	0	0 (0%)	10 (71.4%)	3 (21.4%)	1 (7.1%)
	21	4 (28.6%)	7 (50.0%)	3 (21.4%)	0 (0%)

**Table 8:** Quality of life (EORTC QLQ-C30) day 0–day 21–day 84.

EORTC QLQ-C30	Day	EA		HB		VitB		Placebo		Sum	
scale		Mean	SD	Mean	SD	Mean	SD	Mean	SD	Mean	SD
Physical functioning	0	72.4	22.2	61	22.4	68.4	15.8	75.3	15.6	69.7	19.4
	21	84.4	13.2	66.7	24.5	75.1	12.7	87.5	10.2	79.1	17.3
	84	82.3	12.2	67.3	26.5	80.5	15.7	85.6	11.2	79.5	17.9

Role functioning	0	58.3	34.4	38.5	30.7	37.8	27.8	52.9	28.4	47.2	30.8
	21	72.6	26.6	61.5	37.5	58.9	20.8	74.5	23.7	67.2	27.5
	84	64.5	30.3	56.4	31.6	53.3	29.7	71.4	16.2	61.9	27.4

Emotional functioning	0	63.1	21.6	44.2	25.3	54.4	27.1	62.3	26.4	56.5	25.7
	21	72.6	15.1	62.2	28.0	66.7	24.6	70.4	27.3	68.2	24.1
	84	70.6	18.6	52.3	35.5	62.2	32.7	72.0	19.1	64.8	27.5

Cognitive functioning	0	66.7	23.6	39.7	30.1	62.2	28.5	67.6	24.6	59.9	28.2
	21	70.2	18.7	55.1	30.0	71.1	24.0	79.4	18.2	69.8	23.9
	84	76.7	21.8	58.5	36.2	61.6	25.8	71.0	13.2	67.2	25.2

Social functioning	0	66.7	32.0	50.0	34.0	46.7	34.6	63.7	27.8	57.1	32.3
	21	83.3	17.3	62.8	36.1	60.0	32.0	77.5	25.6	71.2	29.3
	84	81.9	20.3	60.8	40.6	64.7	26.9	80.2	21.4	72.4	28.6

Global health	0	58.9	12.9	55.1	21.7	51.7	20.7	58.3	17.7	56.1	18.2
	21	67.9	13.4	62.8	26.0	61.7	17.2	67.6	17.4	65.1	18.5
	84	71.0	12.4	59.1	27.0	64.7	14.3	72.4	15.0	67.1	18.0
